# Primary intraosseous hybrid epithelioid schwannoma/perineurioma in the proximal tibia: a case report of benign hybrid neoplasm with local hypercellularity

**DOI:** 10.1186/s13000-019-0829-x

**Published:** 2019-06-01

**Authors:** Yuejiao Lang, Dawei Liu, Pei Xiang, Jilin Wang, Yang Li

**Affiliations:** 1 Department of Pathology, The First Affiliated Hospital, Sun Yat-sen University, Guangzhou, Guangdong 510000 People’s Republic of China; 2 Department of Medical Imaging, The First Affiliated Hospital, Sun Yat-sen University, Guangzhou, Guangdong 510000 People’s Republic of China; 3 Department of Pathology, Central People’s Hospital of Zhanjiang, Zhanjiang, Guangdong 524000 People’s Republic of China

**Keywords:** Hybrid peripheral nerve sheath tumours, Epithelioid schwannoma, Perineurioma, Intraosseous neoplasm, Hypercellularity

## Abstract

**Background:**

As a new entity included in the 4th edition of the WHO classification of tumours of soft tissue and bone in 2013, hybrid peripheral nerve sheath tumours are benign composite neoplasms that demonstrate features of more than one type of nerve sheath tumour, with a wide age distribution and a predilection for superficial location. Those involving deep sites are relatively rare. To the best of our knowledge, only one case of primary intraosseous hybrid peripheral nerve sheath tumours has been documented. In this article, we report another case of hybrid peripheral nerve sheath tumours occurring in bone with different clinical, radiological and pathological features from those in the previously reported cases.

**Case presentation:**

A 28-year-old female presented with a painful nodule in the right tibia. Radiological examination revealed an oval eccentric osteolytic lesion in the proximal tibia. Histologically, the circumscribed but unencapsulated lesion demonstrated biphasic cellular differentiation. Bland, small epithelioid cells arranged in clusters in the myxoid or collagenous stroma and inconspicuous spindle cells scattered in the hypercellular areas were suggested to originate from Schwann cells according to the detection of S100. Both the elongated spindle cells with thin, wavy nuclei and the spindle cells in fascicular or storiform pattern in hypercellular areas showed a positive immunoreaction for epithelial membrane antigen, indicating perineurial differentiation. Based on histological and immunochemical examinations, the patient was diagnosed with hybrid epithelioid schwannoma/perineurioma. The lesion was resected and has not recurred for 8 months since resection.

**Conclusion:**

The present case is the second primary intraosseous hybrid peripheral nerve sheath tumour to be reported. This is also the first reported intraosseous tumour composed of epithelioid schwannoma and perineurioma with hypercellularity, indicating diverse involvement sites and a wide range of histological features among hybrid peripheral nerve sheath tumours. Awareness of such diversity is critical for accurate diagnoses. The morphological overlap with other spindle and epithelioid cell neoplasms, especially pure peripheral nerve sheath tumours, requires that immunochemical and molecular examinations be used as objective tools to provide the necessary information for a differential diagnosis.

**Electronic supplementary material:**

The online version of this article (10.1186/s13000-019-0829-x) contains supplementary material, which is available to authorized users.

## Introduction

Peripheral nerve sheath tumours encompass a spectrum of well-defined clinicopathological entities, ranging from benign tumours, such as schwannomas, neurofibromas and perineuriomas, to high-grade malignant neoplasms [1]. Schwannomas constitute a group of peripheral nerve sheath tumours composed of differential neoplastic Schwann cells with variable morphological properties. Epithelioid schwannoma, a relatively uncommon variant, consists of epithelioid or polygonal Schwann cells with small and bland nuclei arranged in clusters or in a linear pattern in collagenous or myxoid stroma. Diffuse and strong staining for S100 protein is characteristic to all morphological variants [2]. Perineuriomas are benign neoplasms with advanced perineurial differentiation and are classified into two types: intraneural and soft tissue perineuriomas [1]. Soft tissue perineuriomas typically present with bland, elongated cells in storiform, lamellar or fascicular pattern. The nuclei vary from wavy and thin to round and pale. Some areas could be quite hypercellular; however, the nuclei are cytologically bland, and mitotic figures are usually rare or absent. Most cases show positive staining for epithelial membrane antigen (EMA) [2].

Occasionally, neoplasms exhibit the co-existence of more than one conventional type of nerve sheath tumour and are difficult to fit into one specific diagnostic category. Such tumours were included in the 4th edition of the WHO classification of tumours of soft tissue and bone in 2013 as a new entity named hybrid peripheral nerve sheath tumours (HPNSTs). According to the histological components, a HPNST can be further classified into hybrid schwannoma/neurofibroma, schwannoma/perineurioma, neurofibroma/perineurioma and schwannoma/neurofibroma/perineurioma [3]. Although some series of cases have been reported [4–6], the incidence might be largely underestimated. These tumours occur over wide age range with a peak in young adults and can arise in a wide distribution of anatomical locations, most commonly in the dermis and subcutis. To our knowledge, there has been only one case of primary intraosseous HPNSTs [7]. In this article, we report the case of a patient with hybrid epithelioid schwannoma/perineurioma in the tibia showing distinct clinical and histological features. The pathological differential diagnoses are discussed. The present case suggests diversity in the morphological and immunohistochemical characteristics and anatomical locations of HPNSTs.

## Materials and methods

The tissue obtained via curettage was processed using routine histological methods: 10% formalin fixed, paraffin embedded and haematoxylin-eosin stained. Both immunohistochemical studies and fluorescence in situ hybridization (FISH) analysis were carried out on formalin-fixed paraffin-embedded (FFPE) tissue. Appropriate positive and negative controls were applied simultaneously. The primary antibodies used for the immunohistochemical studies are listed in Table 1. FISH analysis was performed to evaluate SYT (SS18) gene rearrangements. Briefly, the 5′- and 3′- terminal regions of the SYT gene were labelled with orange and green fluorescent probes (GP Medical Technologies, Ltd., Beijing, China), respectively. One hundred consecutive nuclei showing complete (two orange and two green) signals were scored with the threshold of 15% break-apart signals set as a positive result. Nuclei with incomplete signals were omitted.

**Table 1:** Proteins explored in the present study

Targeted proteins	Clone	Dilution	Expression
Vimentin^a^	V9	1:400	+
CK^a^	AE1/AE3	1:300	–
S100^a^	Polyclonal	1:800	+
Collagen IV^b^	CIV22	1:100	Focally+
SOX10^b^	JG339	1:300	–
EMA^a^	GP1.4	1:800	+
Ki-67^a^	MIB-1	1:800	< 1%
STAT-6^b^	GR500	1:800	–
CD34^a^	QBEnd/10	1:200	–
CD99^a^	12E7	1:300	+
Bcl-2^a^	bcl-2/100/D5	1:400	Patchily +
Actin^a^	1A4	1:200	Focally +

^a^: Dako cytomation, Denmark; ^b^: ZSGB-Bio, China

## Case presentation

### Clinical history

A 28-year-old female presented with a palpable and painful nodule that had been present for five years in the right tibia. The pain was gradually aggravated in the last four months. On the day of presentation, the pain was so unbearable that it limited the movement of the right lower limb. Physical examination revealed a tenderness along the medial portion of the right proximal tibia. There was no external wound, bone friction or rubbing, swelling of the right popliteal lymph nodes or circulation disturbances. Clinical examination failed to show any features of neurofibromatosis, and her familial history was uneventful. Lesion resection and artificial bone graft were performed successively. The patient had a disease-free follow-up 8 months after the surgery.

### Radiological examination

**Fig. 1:**
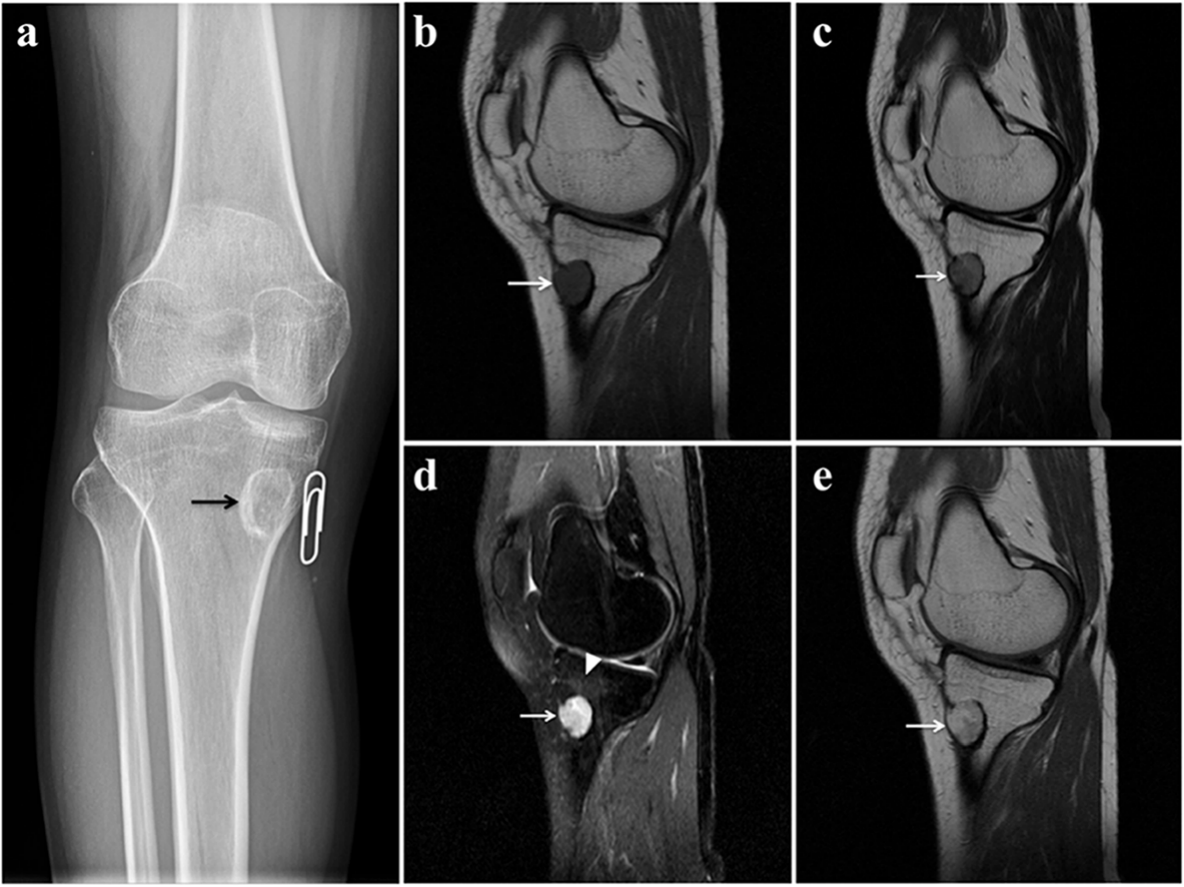
Radiological features of the lesion. **a**. A conventional anterior-posterior radiograph revealed an osteolytic lesion located in the medial superior metaphysis of the right tibia. The sclerotic rim (black arrow) indicated non-aggressiveness. **b**-**e**. Magnetic resonance imaging demonstrated oval eccentric osteolytic lesions in the right tibial medial condyle. It appeared non-specifically isointense on T1WI (**b**), hyperintense on T2WI, both without (**c**) and with (**d**) fat suppression, and enhanced heterogeneously on contrast-enhanced T1WI (**e**). There was endosteal scalloping and cortical expansion but no cortical disruption, periosteal reaction or soft-tissue mass (white arrow), suggestive of non-aggressiveness. Peritumoural oedema (arrowhead) was noted on T2WI with fat suppression (**d**)

A conventional anterior-posterior radiograph showed an osteolytic lesion, 2.0*1.6*1.4 cm in size, located in the medial superior metaphysis of the right tibia. The sclerotic rim was observed, indicating its non-aggressiveness (Fig. 1a). Magnetic resonance imaging demonstrated an oval eccentric osteolytic lesion in the right tibial medial condyle, with endosteal scalloping and cortical expansion but no cortical disruption, periosteal reaction or soft-tissue mass, which are also features that suggest non-aggressiveness (Fig. 1b-e).

2 months after the surgery, no recurrence was identified by the conventional radiograph (data not shown).

### Pathological, immunohistochemical and molecular features

**Fig. 2:**
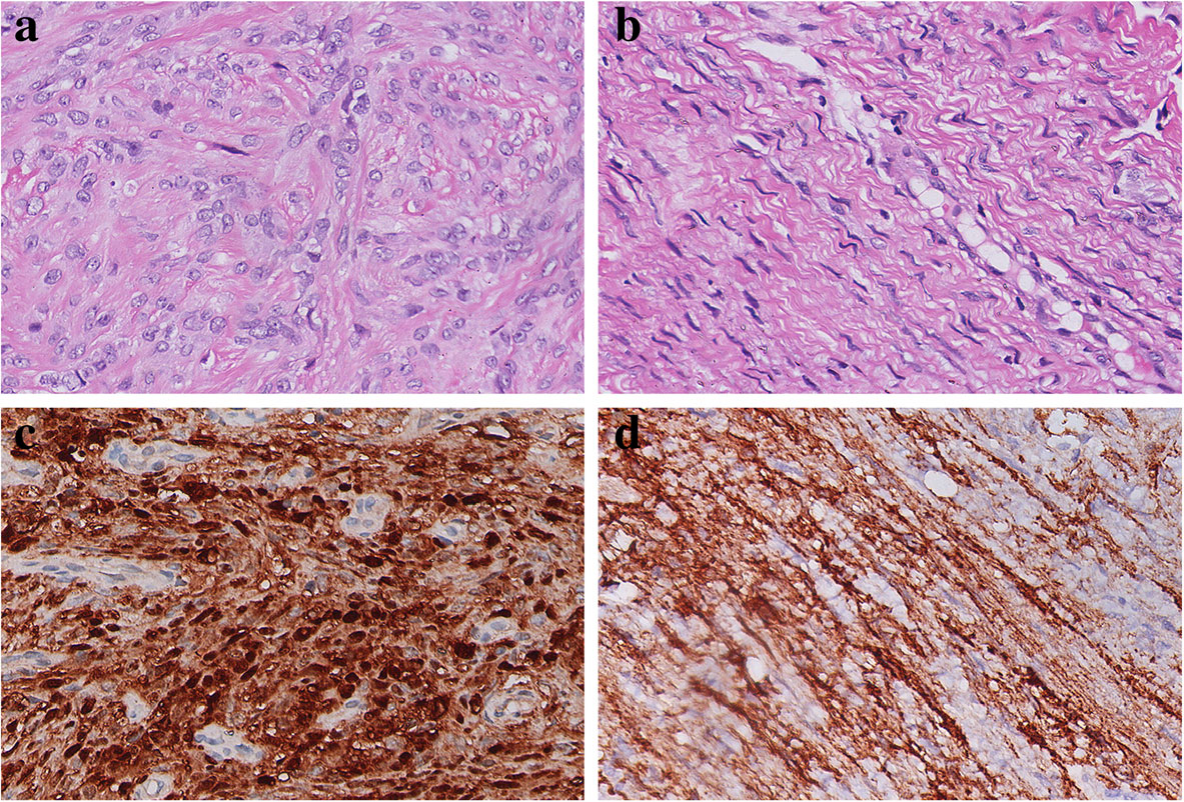
Histological and immunohistochemical characteristics of the hypocellular areas. **a**. Epithelioid cells with bland nuclei and eosinophilic cytoplasm arranged in small clusters within fibromyxoid matrix. Haematoxylin-eosin staining, 400x magnification. **b**. Elongated spindle cells arranging in a lamellar architecture harboured wavy, thin nuclei and delicate bipolar cytoplasmic processes. Haematoxylin-eosin staining, 400x magnification. **c**. Epithelioid cells showed a nuclear and cytoplasmic positive reaction for S100 protein. Immunohistochemical staining, 400x magnification. **d**. Elongated spindle cells exhibited a positive reaction for EMA. Immunohistochemical staining, 400x magnification

**Fig. 3:**
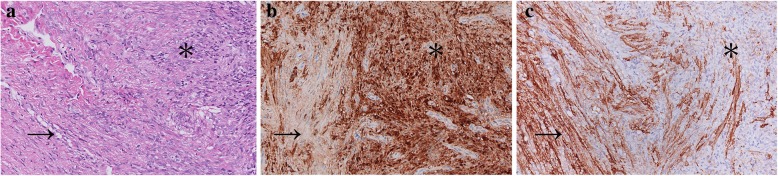
The boundaries of two morphological components in hypocellular areas. **a**. The epithelioid cells (black asterisk) and elongated spindle cells (black arrow) composed of the hypocellular areas. Haematoxylin-eosin staining, 200x magnification. b-c. The blurred boundaries of two morphological areas were demonstrated by non-overlapping staining of S-100(**b**) and EMA(**c**) in the same field as Fig. 3a. Immunohistochemical staining, 200x magnification

**Fig. 4:**
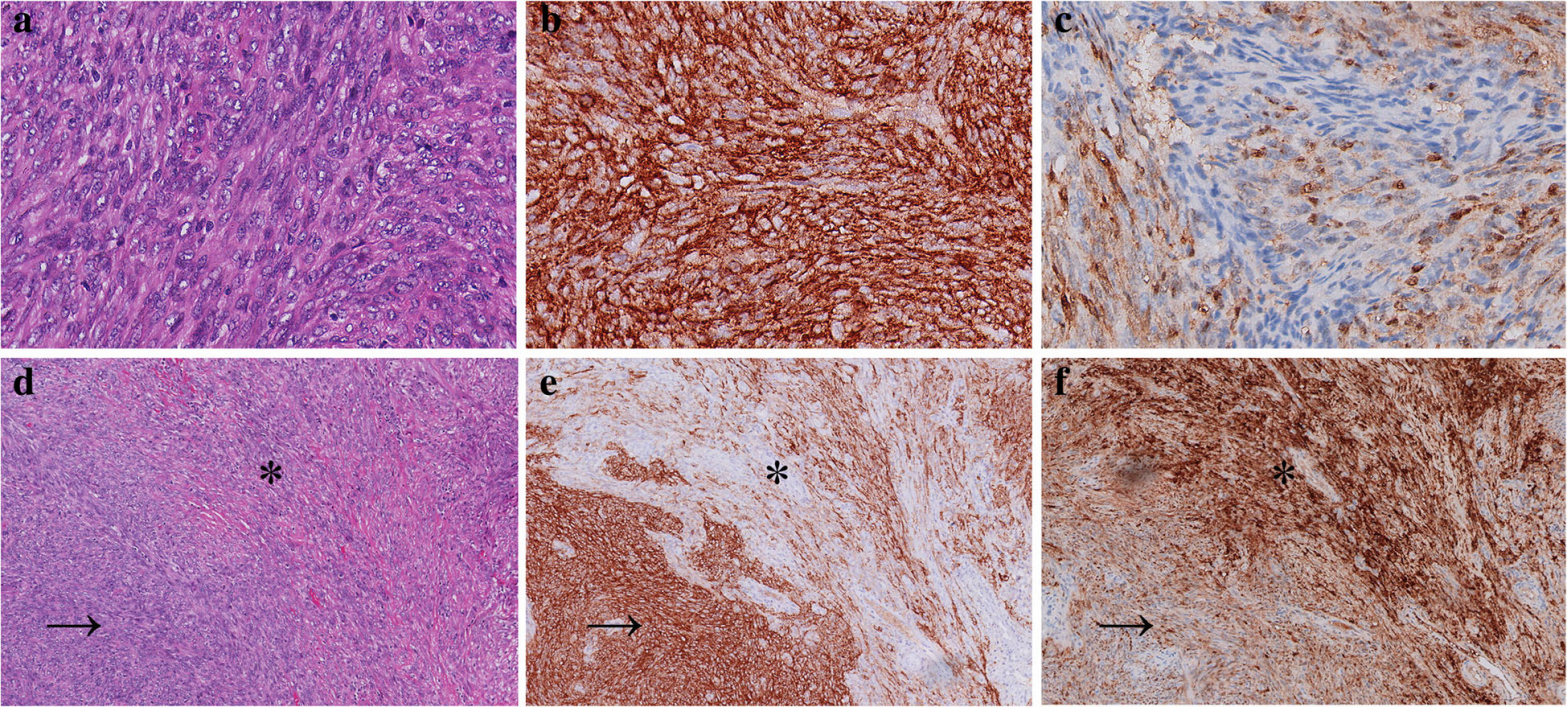
Histological and immunohistochemical characteristics of the hypercellular areas. **a**. The spindle cells arranged in a fascicular or storiform growth pattern. Focally, cells have a streaming or syncytial pattern. Haematoxylin-eosin staining, 400x magnification. b-c. EMA (**b**) and S100(**c**) expression was detected. Most spindle cells showed EMA+/S100- staining, only scattered cells exhibited the opposite expression pattern. Immunohistochemical staining, 400x magnification. **d**. The hypercellular (black arrow) areas and the surrounding hypocellular areas(black asterisk) formed a vague boundary at low magnification. Haematoxylin-eosin staining, 200x magnification. **e**-**f**. The boundaries between hypercellular (black arrow) and hypocellular (black asterisk) area were demonstrated by non-overlapping expression of EMA (**e**) and S100 (**f**) in the same field as Fig. 4d. Immunohistochemical staining, 200x magnification

Gross examination showed solid tissues with firm, white cut surfaces, measuring 2 cm in aggregate. Histologically, low-power magnification revealed a well-circumscribed and unencapsulated lesion with variable cellularity. The relatively hypocellular areas, which was the predominant part of the neoplasm, presented with biphasic cellular morphology. One morphology of these areas exhibited epithelioid cells with bland nuclei and eosinophilic cytoplasm arranging in small clusters within the myxoid or collagenous matrix (Fig. 2a). The other showed cells bearing wavy and thin nuclei and elongated cytoplasmic processes in a lamellar pattern (Fig. 2b). On the immunohistochemical examination, the epithelioid cells were diffuse positive for S100 protein (Fig. 2c), focally positive for Collagen IV (Additional file 1: Figure S1), but negative for EMA. The cells with slender nuclei and elongated cytoplasmic processes stained positively for EMA in a membranous pattern (Fig. 2d), but were negative for S100 or Collagen IV. These two different morphological areas alternated with each other and exhibited blurred boundaries (Fig. 3a-c). Additionally, in some areas composed of epithelioid cells, several hypercellular nodules with distinct morphologies were observed. These vague nodular structures consisted of spindle cells arranged in a fascicular or storiform architecture. Focally, the cells exhibited a streaming or syncytial pattern (Fig. 4a). Immunohistochemically, most spindle cells were diffuse and uniformly positive for EMA (Fig. 4b) but negative for S100 (Fig. 4c); only a minority of cells displayed the opposite expression pattern. Due to the increased nuclear density, these vague nodular structures imparted an overall blue to purple colour with staining at low magnification (Fig. 4d). The abrupt transition between these hypercellular spindle cells areas and surrounding epithelioid cells areas were well demonstrated by the non-overlapping expression of EMA and S100(Fig. 4e-g). Most cells were positive for CD99, and the expression seemed to be stronger in EMA positive areas than in S100 positive areas (Additional file 2: Figure S2). Small-to-mid-sized blood vessels with hyalinized walls were scattered throughout the neoplasm. Lymphoid infiltration was also observed and was most prominent around the vessels. Necrosis, haemorrhage and atypical mitosis were absent, the mitotic activity was very low ranging from 0 to 2 per 50 HPF in the lesion, indicating its benign nature. The Ki-67 index was less than 1% throughout the neoplasm. Expression levels of other proteins explored in the study are listed in Table 1. FISH analysis targeting the rearrangement of SYT was negative (Additional file 3: Figure S3).

Based on the above findings, the present case was diagnosed as hybrid epithelioid schwannoma/perineurioma primarily occurring in the right tibia.

## Discussion and conclusions

Most benign peripheral nerve sheath tumours showing distinctive morphological and immunochemical features can be classified into a certain category. In 1998, Feany et al. [8] first reported nine cases of neurofibroma with Schwann cell differentiation. Since then, cases displaying hybrid characteristics of more than one type of conventional benign nerve sheath tumour have been reported successively and have been named HPNSTs. Patients with HPNSTs frequently present as with a single, slow-growing painless or painful mass with the largest diameter up to 18 cm [9].

Hybrid schwannoma/perineurioma is the most common type of HPNSTs [2]. Due to the morphological overlap with pure nerve sheath tumours, the diagnosis usually requires immunohistochemistry. Strong S100 expression in a large number cells reflects a schwannoma-like component. EMA is the representative marker for and stains the majority of perineuriomas, typically in a membranous fashion [10]. Based on the histological and immunochemical findings, we confirmed the proliferation of neoplastic Schwann cells with S100 positivity and EMA-positive perineurial cells in the present case. Some tumour regions showed clearly distinct areas with one of the two components, and other areas consisted of a mixture of both types of cells. Each component harboured more than one type of histological pattern. For the schwannoma-like component, two different appearances were observed, including bland, small cells that are similar to those in epithelioid schwannoma and inconspicuous spindle cells scattered in hypercellular areas. The hallmark of conventional schwannoma, such as the variable admixture of cellular Antoni A and hypocellular Antoni B areas or the formation of Verocay body was absent. Since most schwannoma-like components in HPNSTs reported so far demonstrated characteristic of conventional schwannoma [8, 11–14],the epithelioid schwannoma-like areas observed in the present case seemed to be rare. The other two cases of HPNSTs with such morphologic features involved in the superficial dermis [15, 16].For the perineurial element, the most areas exhibited elongated, spindle-shaped cells with thin and wavy nuclei that are frequently observed in perineurioma. Additionally, the spindle cells were arranged in storiform and fascicular growth patterns that composed the hypercellular areas, and have also proven to be perineurial in origin, according to the detection of EMA.

Compared with S100 and EMA, there were fewer researches on other proteins in HPNSTs that might be expressed in pure schwannoma or perineurioma. Apart from S100, SOX-10 has also been indicated to be a nonspecific, but highly reliable marker for schwannoma [17]. A case of spinal nerve hybrid schwannoma/perineurioma with low malignant potential and peculiar intraneural perineurioma component showed immunoreactivity for Sox10 [18]. However, the present case was negative for SOX-10. Collagen-IV would be present around individual cells and small groups of cells in most epithelioid schwannoma [2], which was identified focally in the epithelioid schwannoma-like areas in the present case, similar to the case reported by Wang L et al. [16]. The S100+/EMA- cells in the hypercelluar areas were negative for Collagen-IV. Although CD34 would be positive in up to 60% perineurioma and some HPNSTs [2], it was negative in our case or the HPNSTs reported by Hornick JL et al. [6]. CD99 was positive in a cell membrane staining pattern throughout the neoplasm, the expression was stronger in perineurioma-like areas than in schwannoma-like areas. It was also reported to be positive in several cases of sclerosing perineurioma [19–21] and a case of intraosseous microcystic/reticular schwannoma in the mandible [22], while negative in a case of epithelioid schwannoma [23]. However, there is no report concerning CD99 expression in HPNSTs, more researches are needed to explore its diagnostic value. In conclusion, these results indicated the immunophenotypic variability in HPNSTs.

HPNSTs have a predilection of superficial locations [1]. Rare cases have presented in the retroperitoneum [24], posterior mediastinum [14], pleura [9], lymph node [25] and colon [26]. In 2014, Chow, L. T. reported the first case of primary intraosseous HPNSTs occurring in the right femur of an 18-year-old man [7]. To the best of our knowledge, the present case is the second one reported. There are clinical and pathological differences between the two patients. Clinically, the patient in Chow, L. T.’s report suffered from a pathological fracture after two contusion injuries in the same region. The pain caused by the first impact subsided but the patient’s condition deteriorated rapidly after the second collision. In the present report, the disease course was chronic (5 years), and the pain gradually aggravated the patient without an obvious reason. Histologically, the tumour reported by Chow, L. T. exhibited the characteristics of conventional schwannoma and perineurioma, and aneurysmal bone cyst formation was simultaneously identified. Morphological features of an epithelioid schwannoma and of conventional and cellular perineuriomas were observed in the present lesion.

Apart from bland pathological characteristics, radiological features such as sclerotic rim indicated non-aggressiveness of the present lesion. The patient was still alive without recurrence 8 months after the surgery. To date, the vast majority of HPNSTs have demonstrated mild morphological features and benign biological behaviour, and local recurrence is extremely rare after standard surgical resection. In 2011, Rekhi et al. reported a case of malignant peripheral nerve sheath tumours (MPNSTs) arising in a hybrid schwannoma/perineurioma, occurring in the thigh of a young male. In some sections of the lesion, the hypercellular neoplasm exhibited diffuse moderate atypia, prominent mitoses, epithelioid malignant change, discrete tumour necrosis and apoptotic debris. p53 and MIB1 (a proliferation marker) immunostaining highlighted 40–50% and 30–40% of the tumour cell nuclei, respectively [27]. However, the patient was lost to follow-up. In 2013, Tomayoshi Hayashi et al. reported one case of spinal nerve hybrid schwannoma/perineurioma with low malignant potential that recurred five months after the first surgery. Both the primary and recurrent tumours showed a high mitotic rate and a Ki-67 index over 10% [18]. This finding might indicate a possible association of high proliferative activity, as reflected by mitotic activity and proliferation markers with malignant potential and local recurrence. However, one case is obviously insufficient to draw any conclusion, highlighting the need for more instances with long-term observation. No distant metastasis has been reported yet.

The main pathological differential diagnoses of the present case included epithelioid schwannoma, perineurioma, MPNSTs and synovial sarcoma (SS), low-grade fibromyxoid sarcoma(LGFMS) and solitary fibrous tumour(SFT). The non-overlapping S100 and EMA positive cell components were consistent with the hybrid features and suggest evidence against the diagnosis of pure epithelioid schwannoma or perineurioma.

The tumour that perhaps most closely resembles the present case is MPNSTs. MPNSTs are malignant neoplasms showing nerve sheath cellular differentiation and may originate from pre-existing benign PNSTs, from peripheral nerves or within the context of NF1 [2]. The morphological spectrum is relatively broad, varying from well-differentiated areas with an irregular, buckled shape that is characteristic of Schwann cells to less-differentiated areas. Only a few cases of primary intraosseous MPNSTs have been reported in published studies [28], and MPNSTs with hybrid components are extremely rare [18, 27]. Although the hypercellular areas in our case might lead to confusion with MPNSTs, the histological features, including mitoses, nuclear atypia, cellular pleomorphism, necrosis and perivascular tumour cell accentuation, that prompt a malignant diagnosis were not identified in our case. Immunohistochemically, the diffuse and strong positive staining for EMA, the focally positive staining for S100 and the low Ki-67 index (less than 1%) do not support the diagnosis of MPNSTs.

Monophasic SS is a malignant neoplasm with frequent local recurrence and metastasis. SS usually affects young adults and arises in deep soft tissues of the extremities and trunk. As the most common variant, monophasic SS is composed of uniform spindle cells with scanty cytoplasm arranging in vague fascicles or in dense cellular sheets. Variations in cellularity have been observed in some cases, creating a “marble” appearance [2]. Primary intraosseous SS is rare, and only a few cases have been reported [29–33]. These tumours displayed similar histopathology to their soft tissue counterparts [32]. The variable cellularity and the hypercellular areas in our case show some resemblance of monophasic SS. However, the nuclei in monophasic SS should be more monomorphic and often overlapping. Molecular examinations may be more objective for a definite diagnosis of monophasic SS, which shows a specific chromosomal translocation of t(X;18) (p11.2; q11.2), resulting from fusion of the SYT gene to one of the SSX genes that can be identified through FISH or RT-PCR [1, 2, 34]. EMA and CK expression tend to be focal or patchy, often in a single cell distribution [2]. For the present case, the diffuse and strong staining of EMA and a lack of SYT-SSX fusions are against such a diagnosis.

LGFMS often affects young adults and arises in deep soft tissue of proximal extremities and trunk. Patients usually present as with a slow growing, painless mass with long clinical duration [2]. Although the lesion is well circumscribed, it shows microscopic infiltration of peripheral soft tissue. The classic low-power appearance is that of cellular myxoid zones admixed with hypocellular fibro collagenous zones. The two zones are well delineated from one another [2]. In the present case, the hypocellular areas that composed of epithelioid cells showed fibromyxoid matrix, but there was no distinct boundary or cell density difference between areas with myxoid and collagenous stroma. Increased perivascular cellularity, variable perivascular sclerosis and scattered paucicellular collagen rosettes, which may be seen in a subset of LGFMS, were absent. S100 protein positivity was helpful in supporting its Schwann origin.

SFT is a fibroblastic mesenchymal neoplasm composed of uniform spindle to ovoid fibroblastic cells with myxoid stromal change and varying cellularity [2]. Compared with the present case, cells usually arrange in a “patternless” pattern and stromal collagen should be more abundant in SFT. The characteristically feature of SFT-the prominent branching staghorn vascular pattern was absent. Immunohistochemically, SFT could be positive for STAT6, Bcl-2, CD34, CD99 and actin, usually negative for S100 [2]. Therefore, the immunophenotype of the present case was against the diagnose of SFT.

In summary, we described the first case of intraosseous hybrid epithelioid schwannoma/perineurioma. Awareness of the potential occurrence in unusual locations might enhance the recognition of these hybrid tumours. As for intraosseous neoplasm, radiological examination is a useful tool to identify biological nature. Due to the morphological overlap with a pure peripheral nerve sheath, immunohistochemistry is indispensable for definitive diagnosis. Long-term observation and large-scale studies would better reveal the biological behaviour of HPNSTs.

## Additional files

Additional file 1: **Figure S1**. Collagen-IV was present around individual cells and small groups of cells in the hypocellular areas composed of epithelioid cells. Immunohistochemical staining, 400x magnification. (TIF 8519 kb)

Additional file 2: **Figure S2**. The epithelioid cells(a), the elongated spindle cells(b) and the spindle cells composed of hypercellular areas(c) were positive for CD99. Immunohistochemical staining, 400x magnification. (TIF 8660 kb)

Additional file 3: **Figure S3**. The tumour showed a normally fused SYT signal. A broken SYT signal was not detected by fluorescence in situ hybridization. (orange, the 5′- terminal region of the SYT gene; green, the 3′- terminal region of the SYT gene). (TIF 5591 kb)

## Abbreviations

EMA: Epithelial membrane antigen
FFPE: Formalin-fixed paraffin-embedded
FISH: Fluorescence in situ hybridization
HPNSTs: Hybrid peripheral nerve sheath tumours
LGFMS: Low-grade fibromyxoid sarcoma
MPNSTs: Malignant peripheral nerve sheath tumours
SFT: Solitary fibrous tumour
SS: Synovial sarcoma
