# Supplementary figures and images for: Primary intraosseous hybrid epithelioid schwannoma/perineurioma in the proximal tibia: a case report of benign hybrid neoplasm with local hypercellularity
Source: Diagn Pathol. 2019 Jun 1;14:51. doi: 10.1186/s13000-019-0829-x (PMC6545219; doi:10.1186/s13000-019-0829-x)

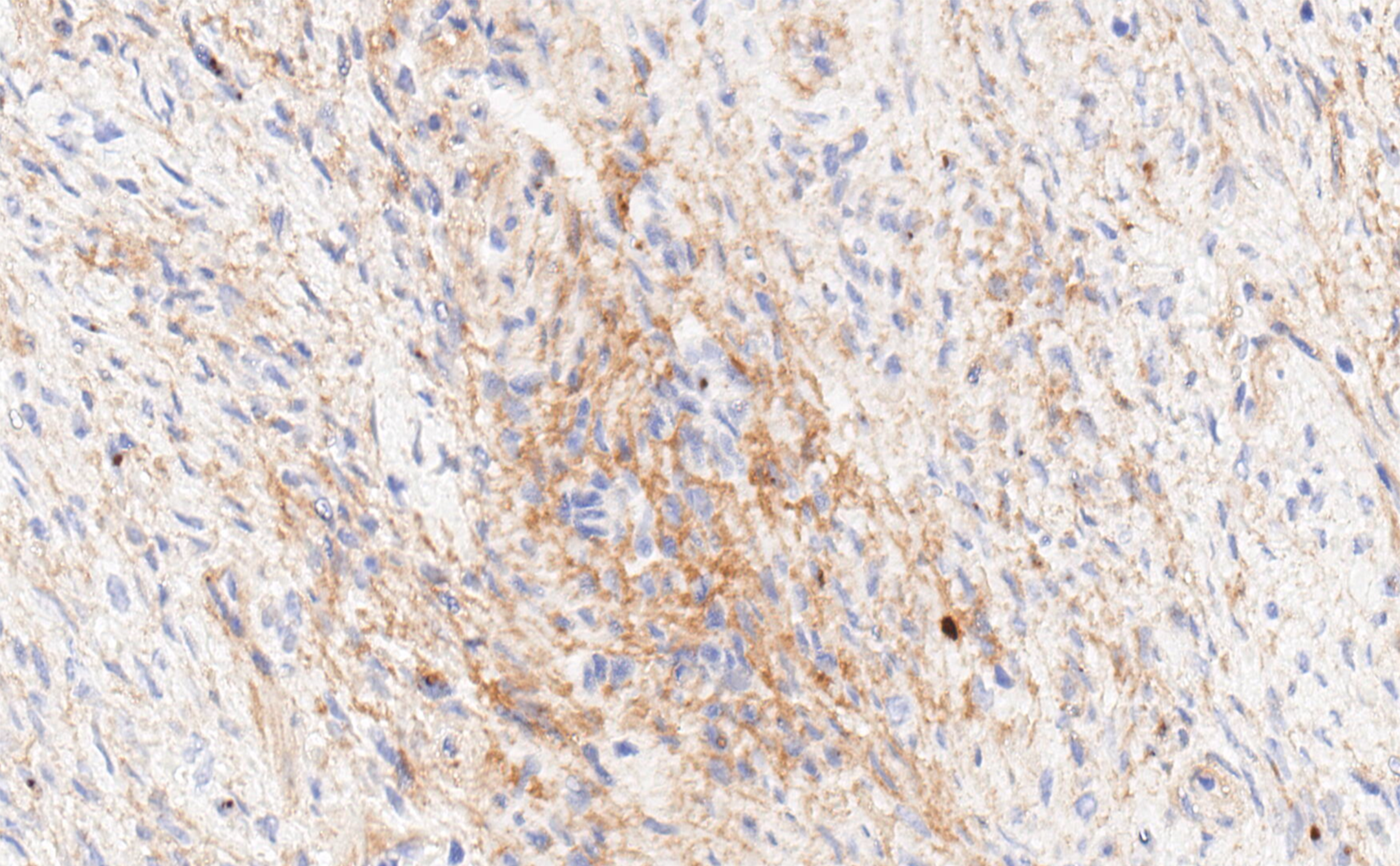

Supplement: Supplementary file 1 — Figure S1. Collagen-IV was present around individual cells and small groups of cells in the hypocellular areas composed of epithelioid cells. Immunohistochemical staining, 400x magnification. (TIF 8519 kb) [file 13000_2019_829_MOESM1_ESM.tif]

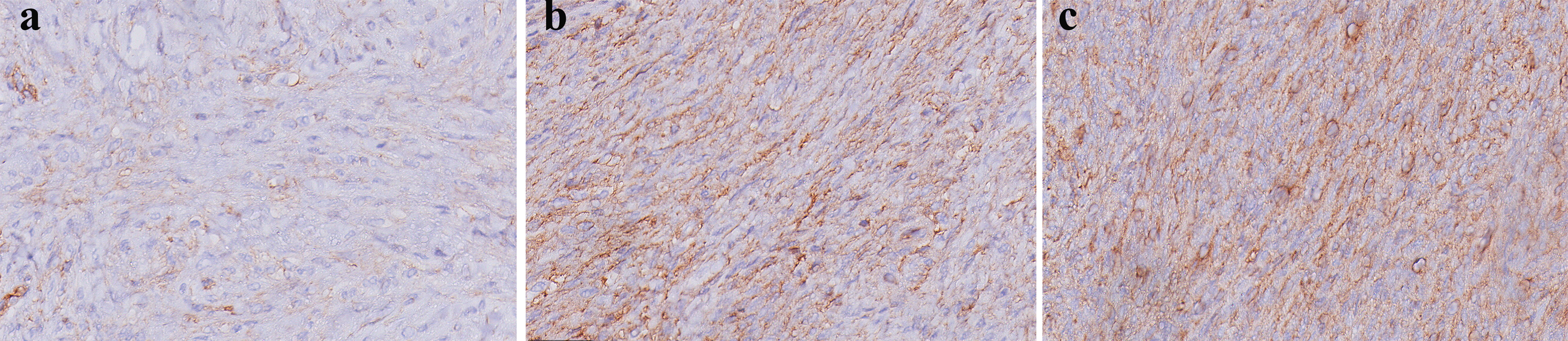

Supplement: Supplementary file 2 — Figure S2. The epithelioid cells(a), the elongated spindle cells(b) and the spindle cells composed of hypercellular areas(c) were positive for CD99. Immunohistochemical staining, 400x magnification. (TIF 8660 kb) [file 13000_2019_829_MOESM2_ESM.tif]

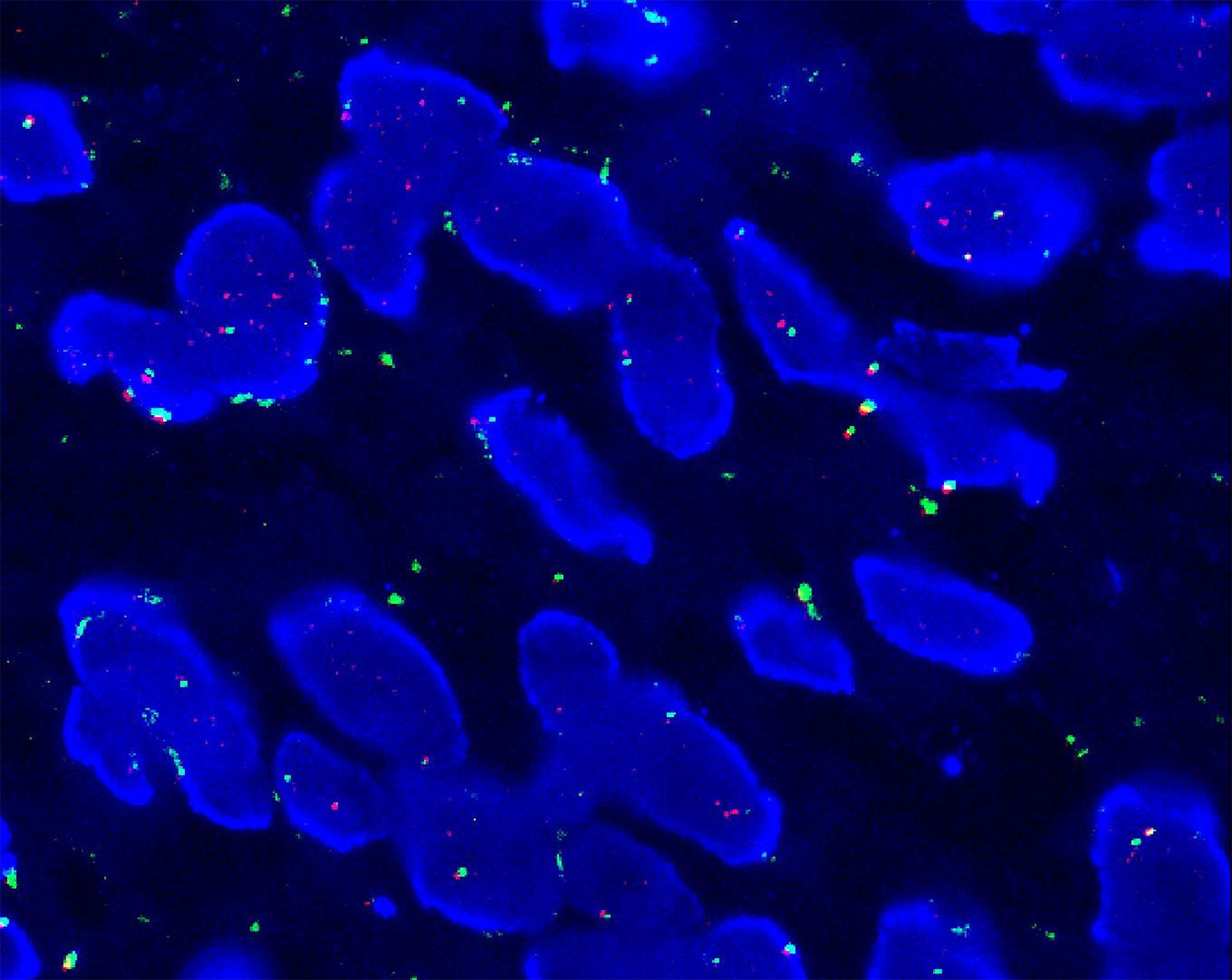

Supplement: Supplementary file 3 — Figure S3. The tumour showed a normally fused SYT signal. A broken SYT signal was not detected by fluorescence in situ hybridization. (orange, the 5′- terminal region of the SYT gene; green, the 3′- terminal region of the SYT gene). (TIF 5591 kb) [file 13000_2019_829_MOESM3_ESM.tif]
